# Clinical Practice, Challenges, and the Future of Ophthalmic Genetics in Saudi Arabia

**DOI:** 10.18502/jovr.v20.15890

**Published:** 2025-04-02

**Authors:** Basamat AlMoallem, Ghadah Alsuwailem, Nadeef Alqahtani, Layan Alshammari, Abeer Alkhodier

**Affiliations:** ^1^Department of Ophthalmology, College of Medicine, King Saud University (KSU), Riyadh, Saudi Arabia; ^2^Department of Ophthalmology, King Saud University Medical City (KSUMC), Riyadh, Saudi Arabia; ^3^College of Medicine, King Khalid University Hospital/King Saud University, Riyadh, Saudi Arabia; ^4^College of Medicine, Imam Mohammed bin Saud Islamic University, Riyadh, Saudi Arabia; ^5^Department of Ophthalmology, King Khaled Hospital, Hail, Saudi Arabia

**Keywords:** Challenges, Genetic Counselling, Ophthalmic Genetics, Recommendations, Referral Pattern, Saudi Arabia

## Abstract

**Purpose:**

Ophthalmic genetics is vital for diagnosing and managing inherited eye disorders, contributing to personalized treatments.

**Methods:**

This cross-sectional study assessed 131 healthcare professionals in Saudi Arabia through an online questionnaire to evaluate clinical practices, referral patterns, and challenges in genetic ophthalmology.

**Results:**

Our study showed that 61.7% of participants reported the availability of ophthalmic genetics services in their hospitals, with an equal percentage referring patients to genetic specialists. However, significant barriers were identified, including limited budgets for genetic testing (69.6%), a lack of trained physicians (70.9%), low community awareness (50.6%), and a perceived lack of treatment options (27.8%). Additionally, concerns about patient access to genetic testing (54.3%) and genetic counseling (50.6%) were highlighted.

**Conclusion:**

This is the first study on ophthalmic genetics in Saudi Arabia, and its findings emphasize the need for policy reforms and targeted interventions. Proposed solutions include innovative financial models for genetic testing, expanded training programs for healthcare providers, and public awareness campaigns to improve access to genetic services. Addressing these challenges can enhance early diagnosis, treatment strategies, and patient outcomes in ophthalmic genetics.

##  INTRODUCTION

Ophthalmic genetics encompasses the diagnosis and treatment of individuals, spanning from children to adults afflicted by genetic eye conditions such as corneal dystrophies, retinitis pigmentosa, and chromosomal disorders.^[1]^ According to the comprehensive analysis of Louise F. Porter and Graeme Black, ophthalmology is a pioneering domain in personalized medicine. This pioneering approach harnesses the power of genomic technologies to enhance molecular diagnostics and provide for individualized patient needs.^[2]^ With the rise of genetic eye diseases due to inbreeding and large families, the pattern of eye diseases in Saudi Arabia has changed significantly in the last few decades.^[3]^

Genetic testing technologies have become more powerful and sophisticated, facilitating the discovery of genes responsible for inherited eye diseases. Over 500 genes contributing to inherited eye diseases have been discovered in the past decades. Genetic testing will play an essential role in managing these disorders in the future.^[4,5]^

The indications for genetic testing can be divided into five broad categories: treatment, diagnosis, prognosis, counselling, and research. However, the American Academy of Ophthalmology recommends genetic testing for disorders where the causative genes are known, especially for patients with disorders that can be treated or prevented, such as inherited retinal degenerations, early-onset glaucoma, and optic neuropathies. Additionally, genetic tests can identify patients and family members at risk.^[6]^

Since this is the first study in the Kingdom to explore the status of genetic ophthalmology, there is limited data regarding challenges or barriers. However, it is well recognized that the subspecialty lacks appropriate exposure among residents and medical trainees. Even with proper exposure and interest, local fellowship programs have limited availability compared to other subspecialties. Therefore, our study aims to explore the current practice, challenges, and future of ophthalmic genetics in Saudi Arabia.

##  METHODS

This observational cross-sectional study was approved by the Institutional Review Board, College of Medicine (E-23-7817) and conducted in Saudi Arabia to provide an overview of ophthalmic genetics in Saudi Arabia, assess the clinical practice of ophthalmic genetics in the country, identify challenges, and implement recommendations to enhance current practice in this field. The sample size comprised a total of 131 healthcare professionals.

We included family physicians, general practitioners, ophthalmologists, ophthalmologists in training, and ophthalmic geneticists currently living in Saudi Arabia and provided informed consent.

This study used non-probability convenience sampling. Data were collected through survey questionnaires distributed randomly on multiple online platforms.

Participants were asked to complete a self-administered electronic questionnaire covering demographic information (age, gender, education, and occupation). The participants were also asked about general information regarding their clinical experiences and challenges in ophthalmic genetics [Supplementary 1].

The study objectives were explained to the participants, and their voluntary consent was obtained before enrolling them. The study adhered to the tenets of the Declaration of Helsinki. Statistical analyses were performed using the R package version 3.6.3. Categorical variables were summarized as counts and percentages. Continuous variables were summarized using mean and standard deviation or median and interquartile range for normal and non-normal variables, respectively. To assess the association between different variables, the chi-square and Fisher exact tests were used. Hypothesis testing was performed at a 5% significance level.

**Table 1:** Demographic factors of the participants

		**Count**	**Column ** ***N*** ** %**
Gender	Male	59	45.0%
	Female	72	55.0%
Nationality	Saudi	124	94.6%
	Non-Saudi	7	5.4%
Region within Saudi Arabia	Central region	84	64.1%
	Eastern region	16	12.2%
	Western region	20	15.3%
	Southern region	8	6.1%
	Northern region	3	2.3%
Current job position	Family Physician or General Practitioner	46	35.14%
	Ophthalmology Consultant	33	25.2%
	Ophthalmic Genetic Specialist	1	0.76%
	Ophthalmologist	3	2.3%
	Ophthalmology Resident	40	30.5%
	Ophthalmology Fellow	5	3.8%
	Clinical Geneticist	3	2.3%
Hospital	University Hospital	65	49%
	Ministry of Health Hospital	41	31%
	Military Forces Hospital	10	8%
	National Guard Hospital	5	4%
	Primary Care Hospital	5	4%
	Private Hospital	4	3%
	Security Force Hospital	1	1%

##  RESULTS

**Figure 1:**
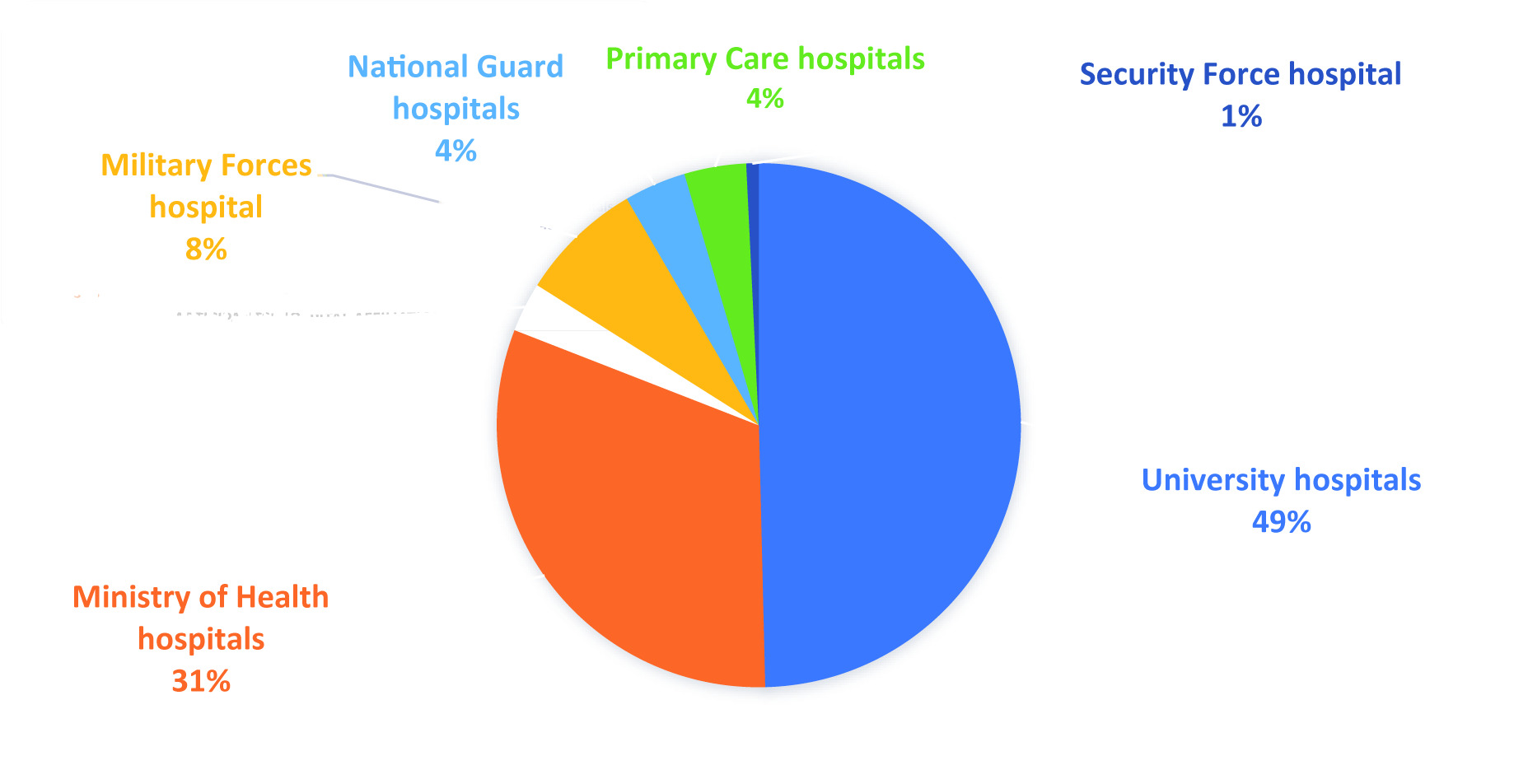
Participants’ hospital affiliations.

The demographic characteristics of the study participants are summarized in Table 1. A total of 131 individuals, including ophthalmologists, ophthalmologists in training, and genetic ophthalmologists currently residing in Saudi Arabia, participated in the study. The gender distribution revealed that 59 (45%) participants were male and 72 (55%) were female. Regarding nationality, most participants were Saudi, accounting for 124 (94.6%), with a smaller proportion being non-Saudi, constituting 7 (5.4%) of the total sample. The participants were dispersed geographically across different areas of Saudi Arabia. With 84 (64.1%) participants, the Central region had the highest representation, followed by the Western (15.3%), Eastern (12.2%), Southern (6.1%), and Northern (3.3%) regions. Participants in the study had a variety of functions in the field of ophthalmology. The largest group consisted of 40 (30.5%) ophthalmology residents, followed by 33 (25.2%) ophthalmology consultants, 5 (3.8%) ophthalmology fellows, 3 (2.3%) clinical geneticists, 3 (2.3%) ophthalmologists, and 1 (0.76%) ophthalmic genetic specialist. The remaining 46 participants were family physicians or general practitioners (35.14%). Participants belonged to a variety of hospital affiliations, with university hospitals accounting for the largest representation (n = 65; 49%), followed by hospitals connected with the Ministry of Health (n = 41; 31%), hospitals affiliated with the Military Forces (n = 10; 8%), and private hospitals (n = 4; 3%). There were five participants from each of the National Guard hospitals (4%), Primary Care hospitals (4%), and Security Forces Hospital (1%) [Figure 1].

The sole ophthalmic genetic specialist in the study, who completed their genetic fellowship at the University of California San Diego (UCSD), has been practicing ophthalmic genetics in Saudi Arabia for over a decade. Despite having attended genetics ophthalmology-related scientific meetings or clubs only once, the specialist has actively participated in a clinical trial for gene therapy and has enrolled patients in clinical gene therapy treatments. The specialist receives referrals primarily from various ophthalmic subspecialties, including anterior segment, pediatric ophthalmology and strabismus, glaucoma, vitreoretinal surgery, medical retina and uveitis, and oculoplastic and orbital surgery. The specialist notes that while the number of genetic ophthalmologists in Saudi Arabia is adequate to meet patient needs, there are challenges in the field, such as the relatively low patient volume compared to other subspecialties and concerns about the average annual income of a practicing genetic ophthalmologist, which is deemed incomparable to other subspecialties. The specialist stresses the need for genetic testing for patients whose clinical findings suggest an inherited disorder, even though their hospital does not have a dedicated ophthalmic genetics clinic. She also advocates for improvements in the patient journey for individuals with inherited ocular diseases. While acknowledging the existence of genetic counselling, the specialist expresses concern regarding patients' access to sufficient counselling.

Regarding hospital practices, the specialist says that patients with clinical signs suggestive of an inherited condition are given genetic testing; however, those with a family history suggestive of an inherited disorder do not receive genetic testing. Estimating 1–2 instances each month, the specialist sees a moderate overall referral rate to genetic ophthalmologists, with the retina specialization playing a major role in these referrals. In terms of hereditary eye diseases, the specialist believes that patients with clinical signs indicative of an inherited disorder are most likely to benefit from genetic testing.

Table 2 sheds light on the accessibility of ophthalmic genetics services, referral trends, and the opinions of medical professionals about patient access to genetic testing and counselling in Saudi Arabia. While 33.3% of respondents said their hospital did not offer an ophthalmic genetics service, the majority (61.7%) stated that their hospital did provide such a service. A negligible portion (4.9%) were uncertain about the accessibility of these services. Furthermore, 61.7% of respondents said they had referred patients to ophthalmic geneticists, but a sizable fraction (49.4%) said that referral frequency was irrelevant. Specifically, 29.6% of those with appropriate recommendations referred patients one to five times a month, 16.0% fewer than that, and 4.9% more than five times.

**Table 2:** Ophthalmic genetics services and access to genetic testing and counselling (*N* = 81)

		**Count**	**Column ** ***N*** ** %**
Is there an Ophthalmic Genetics service in your hospital?	No	27	33.3%
	Yes	50	61.7%
	I do not know	4	4.9%
Have you ever referred a patient to an ophthalmic geneticist?	No	31	38.3%
	Yes	50	61.7%
If yes, how many referrals have you made in a month? (if other, please specify)	Not applicable	40	49.4%
	< 1 time/month	13	16.0%
	1–5 times/month	24	29.6%
	> 5 times/month	4	4.9%
Do you think patients have adequate access to genetic testing?	No	44	54.3%
	Yes	17	21.0%
	Not sure	20	24.7%
Do you think patients have adequate access to genetic counselling?	No	41	50.6%
	Yes	21	25.9%
	Not sure	19	23.5%
Have you attended an ophthalmic genetic workshop or scientific meeting?	No	18	22.2%
	Yes	38	46.9%
	I would attend if available	25	30.9%
Do you offer genetic testing for patients with clinical findings suggestive of an inherited disorder?	No	8	9.9%
	Yes	62	76.5%
	Genetic testing is not available in my area/workplace	11	13.6%

Regarding patient access to genetic services, the opinions of healthcare professionals were as follows: 21.0% thought that patients had sufficient access to genetic testing, whereas 54.3% thought otherwise. Notably, 24.7% of respondents were still unclear about the scope of access. Regarding genetic counselling, 25.9% of respondents were certain that patients had sufficient access, while 23.5% were still unsure. Half of the respondents (50.6%) thought that patients lacked proper access to genetic counselling.

Besides, 46.9% of respondents had attended educational events like scientific symposia or workshops on ocular genetics. A further 30.9% indicated they would want to participate in such events if space was available, and 22.2% had not attended such activities. A majority (76.5%) provided genetic testing for patients whose clinical symptoms suggested inherited illnesses, while 9.9% did not. Only a small portion (13.6%) revealed the unavailability of genetic testing in their area or workplace.

**Figure 2:**
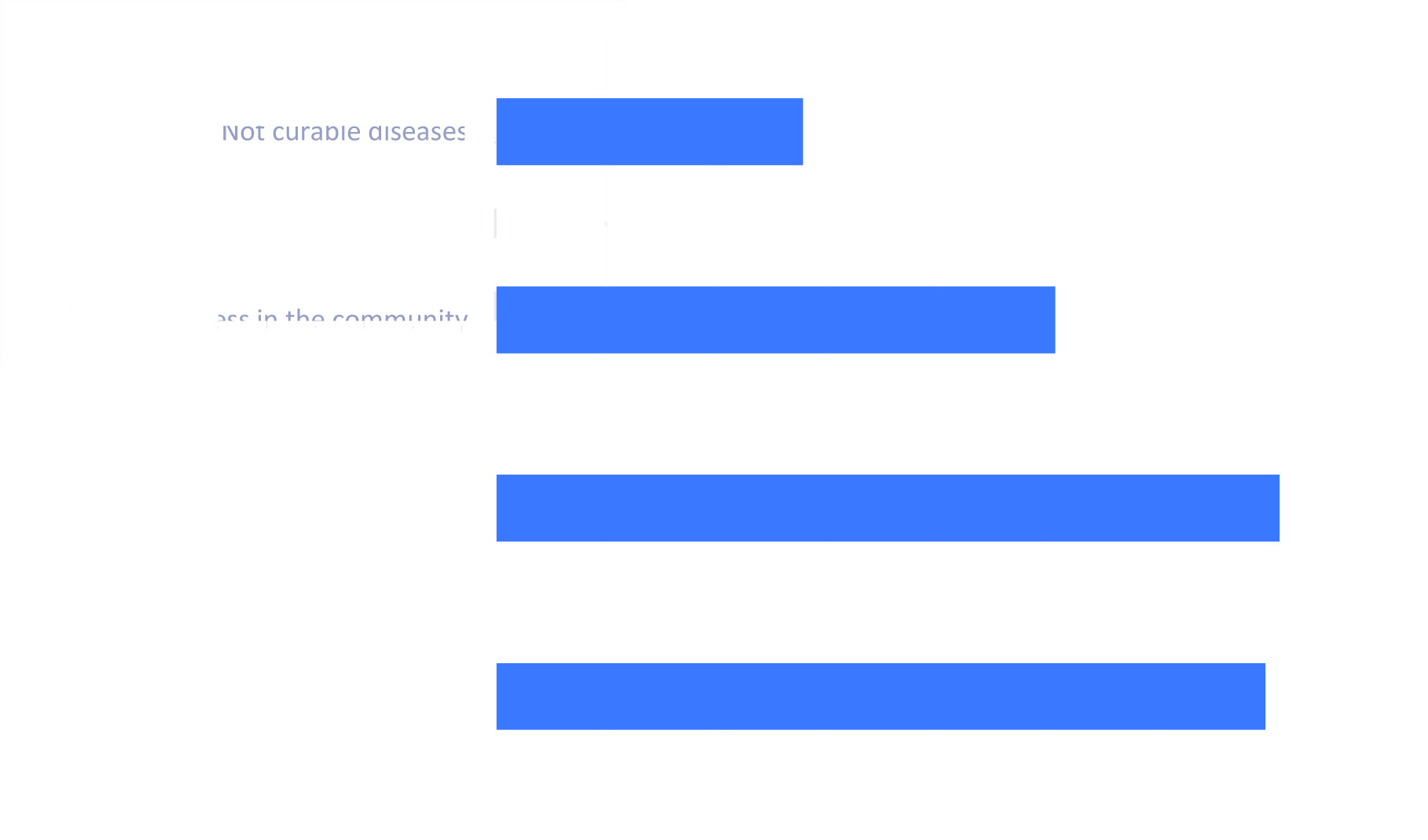
Current challenges to access ophthalmic genetics.

According to Figure 2, access to ophthalmic genetics in Saudi Arabia is challenging. A survey revealed that 70.9% of healthcare professionals cited the lack of trained physicians as the primary obstacle. Additionally, 69.6% of responders identified limited budgets for genetic testing as a major issue, and 50.6% noted low community awareness of ophthalmic genetics as a notable challenge. These barriers hinder the effective delivery of genetic services, highlighting the need for targeted interventions to improve access and education in this critical field.

##  DISCUSSION

Our observational cross-sectional study provided valuable insights into ophthalmic genetics in Saudi Arabia. It examined practitioner demographics, service availability, referral patterns, and access challenges, alongside healthcare professionals' views on genetic testing and counselling. By situating our findings within the broader literature, we aimed to highlight the current state of ophthalmic genetics and its implications for the future of genetic services in the Saudi healthcare system. This exploration is crucial for enhancing understanding and improving practices in this evolving field.

The engagement of professionals in genetic ophthalmology is evident, with ophthalmology residents comprising 30.5% and consultants 25.2% of the sample in the present study. This distribution reflects a commitment to advancing knowledge and practice in the field across various career stages. Additionally, the prevalence of university hospitals as the leading practice setting indicates a robust infrastructure for training and research, which could foster collaboration and innovation in genetic ophthalmology. This diverse representation underscores the importance of integrating genetic insights into ophthalmic care, ultimately enhancing patient outcomes and professional development.

The integration of ophthalmic genetics services in 61.7% of surveyed hospitals marks a significant advancement in ophthalmology in Saudi Arabia. This trend reflects a broader global initiative to embed genetic services within various medical fields. The same percentage of respondents indicated they had referred patients to ophthalmic geneticists. This observation highlights the growing acknowledgment of genetic assessments in treating intricate eye disorders as well as enhancing patient care and outcomes in ophthalmology.

The frequency of referrals for genetic eye disorders reveals significant insights. Nearly half of the practitioners reported non-applicable referrals, suggesting disparities in the prevalence of these disorders and varying levels of awareness regarding genetic testing. Interestingly, 29.6% of respondents indicated they make referrals 1–5 times monthly, highlighting a consistent yet potentially underutilized demand for genetic services. This finding underscores the need for enhanced education and resources to improve referral practices and promote the adoption of genetic testing in managing inherited eye conditions.

The challenges highlighted in Figure 2 reveal significant barriers to accessing ophthalmic genetics services. A major concern is the perception of insufficient funding for genetic testing, which reflects broader economic issues in healthcare globally. This situation underscores the urgent need for healthcare policymakers to investigate innovative funding solutions and enhance insurance coverage. Addressing these financial obstacles can promote equitable access to genetic testing, ultimately improving patient outcomes in ophthalmic genetics.

Moreover, the shortage of trained physicians in genetic services, highlighted by a 70.9% concern rate, reflects a global challenge in developing specialized care. This gap in expertise hinders the effective implementation of precision medicine, as limited knowledge of genomics is a significant barrier. To address this issue, it is essential to establish targeted educational and training programs aimed at enhancing the workforce's capabilities in providing comprehensive genetic services. Such initiatives are crucial for improving patient outcomes and advancing the field of genomics.

The recognition of low awareness in the community (50.6%) aligns with studies that emphasize the pivotal role of public awareness in the success of genetic services.^[12,13]^ Educational campaigns targeting both healthcare professionals and the general public can raise awareness and recognition of the importance of genetic testing in ophthalmology.

The perception that certain eye diseases are not curable (27.8%) highlights the need for ongoing efforts to educate healthcare professionals and the public about the potential benefits of genetic testing, even in the absence of curative treatments. Emerging therapies, including gene therapies, are continually advancing, and understanding the genetic basis of diseases can inform treatment strategies.^[14]^

Access to genetic testing and counselling remains a significant concern in healthcare. Although 76.5% of healthcare providers offer genetic testing for patients with signs of inherited disorders, 13.6% report that such testing is unavailable in their regions or workplace. This disparity highlights the urgent need for improved infrastructure and resources to enhance the accessibility of genetic services. Ensuring all patients can access these vital services is essential for effective diagnosis and management of genetic conditions. Expanding the availability of genetic services will ultimately lead to better patient outcomes and informed healthcare decisions, in addition to improved infrastructure and resources.

Thus far, access to genetic testing and counselling remains a significant challenge, with 54.3% of patients perceiving inadequate access to testing and 50.6% to counselling. These perceptions reflect broader barriers, such as limited awareness and availability of genetic services. To improve access, innovative strategies like telemedicine and partnerships with genetic counselling services are essential. By leveraging technology and collaboration, we can enhance the delivery of genetic care and ensure that patients receive the support they need.

The results of this study justify several recommendations to enhance ophthalmic genetics in Saudi Arabia. Firstly, efforts to address budget constraints should involve collaborations between healthcare providers, insurance companies, and policymakers to establish financial models that support patient access to genetic testing. Secondly, investments in educational programs and training opportunities for healthcare professionals can mitigate the shortage of trained specialists and help foster a workforce equipped with genetic services. Thirdly, public awareness campaigns can dispel misconceptions and improve community understanding of the benefits of genetic testing. This aligns with recommendations from international studies emphasizing the importance of community engagement in genetic services.^[16,17]^ Lastly, consistent with the growing trend of telehealth in genetic services, the integration of telemedicine services can bridge geographical disparities, ensuring that patients across the country have access to genetic counselling and testing.^[18]^

While this study provides valuable insights into the state of ophthalmic genetics in Saudi Arabia, several limitations should be considered. Firstly, the study's cross- sectional design offers a snapshot of the current scenario but does not capture temporal changes or causality relations. Longitudinal studies would be beneficial to track trends and assess the impact of interventions over time. Secondly, the reliance on self-reported data through survey questionnaires may introduce response bias, as participants might provide socially desirable answers or have varying interpretations of the questions. Additionally, recall bias may affect the accuracy of information, particularly when participants are asked about historical events or experiences. The sample size in the present study, while sufficient for an observational research, may limit the ability to detect subtle differences within subgroups. Larger sample sizes and more diverse participant groups could enhance the generalizability and robustness of the study findings. Lastly, the study focused on healthcare professionals' perspectives, and the patient's viewpoints were not directly explored. Including patient perspectives would provide a more comprehensive understanding of the challenges and opportunities in genetic ophthalmology.

In summary, our study is the first to identify challenges in ophthalmic genetics and highlight critical areas for improvement in Saudi Arabia. These include addressing budget constraints, enhancing professional training, raising community awareness, and improving access to genetic testing and counselling. To advance the field, future

initiatives must foster collaboration among healthcare stakeholders, policymakers, and the community, which in turn will contribute to the progress of global genomic medicine.

##  Financial Support and Sponsorship

None.

##  Conflicts of Interest

None.

## References

[B1] Singh M, Tyagi SC (2018). Genes and genetics in eye diseases: A genomic medicine approach for investigating hereditary and inflammatory ocular disorders. Int J Ophthalmol.

[B2] Porter LF, Black GC (2014). Personalized ophthalmology. Clin Genet.

[B3] Alkuraya FS (2010). Saudi ophthalmic genetics research: the local and global impact. Saudi J Ophthalmol.

[B4] Méjécase C, Malka S, Guan Z, Slater A, Arno G, Moosajee M (2020). Practical guide to genetic screening for inherited eye diseases. Ther Adv Ophthalmol.

[B5] Gabriel LA, Traboulsi EI (2011). Genetic diagnostic methods for inherited eye diseases. Middle East Afr J Ophthalmol.

[B6] Wiggs JL (2007). Genetic etiologies of glaucoma. Arch Ophthalmol.

[B7] Alizary A, Ahmad K, Al Bakri A (2023). Parental experience with an ocular genetic counseling services in Saudi Arabia. Saudi J Ophthalmol.

[B8] Stefanicka-Wojtas D, Kurpas D (2023). Personalised medicine-Implementation to the healthcare system in Europe (Focus Group Discussions). J Pers Med.

[B9] Raspa M, Moultrie R, Toth D, Haque SN (2021). Barriers and facilitators to genetic service delivery models: Scoping review. Interact J Med Res.

[B10] Suther S, Goodson P (2003). Barriers to the provision of genetic services by primary care physicians: A systematic review of the literature. Genet Med.

[B11] Schaibley VM, Ramos IN, Woosley RL, Curry S, Hays S, Ramos KS (2022). Limited genomics training among physicians remains a barrier to genomics-based implementation of precision medicine. Front Med.

[B12] Henneman L, Vermeulen E, van El CG, Claassen L, Timmermans DR, Cornel MC (2013). Public attitudes towards genetic testing revisited: Comparing opinions between 2002 and 2010. Eur J Hum Genet.

[B13] Hakim Zada F, Ahmad Azahari AH, Wong SW, Ali A, Ismail NA (2023). Understanding challenges of genetic testing on neuromuscular disorders from the parental lens. J Pers Med.

[B14] Liew G, Michaelides M, Bunce C (2014). A comparison of the causes of blindness certifications in England and Wales in working age adults (16-64 years), 1999-2000 with 2009-2010. BMJ Open.

[B15] Kaphingst KA, McBride CM, Wade C, Alford SH, Reid R, Larson E, et al (2012). Patients’ understanding of and responses to multiplex genetic susceptibility test results. Genet Med.

[B16] Nankya H, Wamala E, Alibu VP, Barugahare J (2024). Community engagement in genetics and genomics research: A qualitative study of the perspectives of genetics and genomics researchers in Uganda. BMC Med Ethics.

[B17] Tindana P, de Vries J, Campbell M, Littler K, Seeley J, Marshall P, et al (2015). ; as members of the H3A Working Group on Ethics. Community engagement strategies for genomic studies in Africa: A review of the literature BMC Med Ethics.

[B18] Ma D, Ahimaz PR, Mirocha JM, Cook L, Giordano JL, Mohan P, et al (2021). Clinical genetic counselor experience in the adoption of telehealth in the United States and Canada during the COVID-19 pandemic. J Genet Couns.

